# Effective methods for increasing coumestrol in soybean sprouts

**DOI:** 10.1371/journal.pone.0260147

**Published:** 2021-11-18

**Authors:** Tomoe Ohta, Takuhiro Uto, Hiromitsu Tanaka

**Affiliations:** 1 Faculty of Pharmaceutical Sciences, Department of Pharmacognosy, Nagasaki International University, Sasebo, Nagasaki, Japan; 2 Faculty of Pharmaceutical Sciences, Department of Molecular Biology, Nagasaki International University, Sasebo, Nagasaki, Japan; Presidency University, INDIA

## Abstract

Coumestrol (CM), a biologically active compound found in *Leguminosae* plants, provides various human health benefits. To identify easy and effective methods to increase CM content in vegetables, we developed a quantitative analysis method using high-performance liquid chromatography (HPLC). Using this method, we found that soybean sprouts (1.76 ± 0.13 μg/g) have high CM contents among nine vegetables and evaluated the difference in CM contents between two organs of the sprouts: cotyledons and hypocotyls. Next, soybean sprouts were cultivated under different light, temperature, and water conditions and their CM contents were evaluated. CM content was higher in hypocotyls (4.11 ± 0.04 μg/g) than in cotyledons. Cultivating soybean sprouts at 24°C enhanced CM content regardless of light conditions, the growth of fungi and bacteria, and sprout color. Thus, we identified methods of soybean sprout cultivation to increase CM content, which may provide health benefits and enhance value.

## Introduction

**Fig 1:**
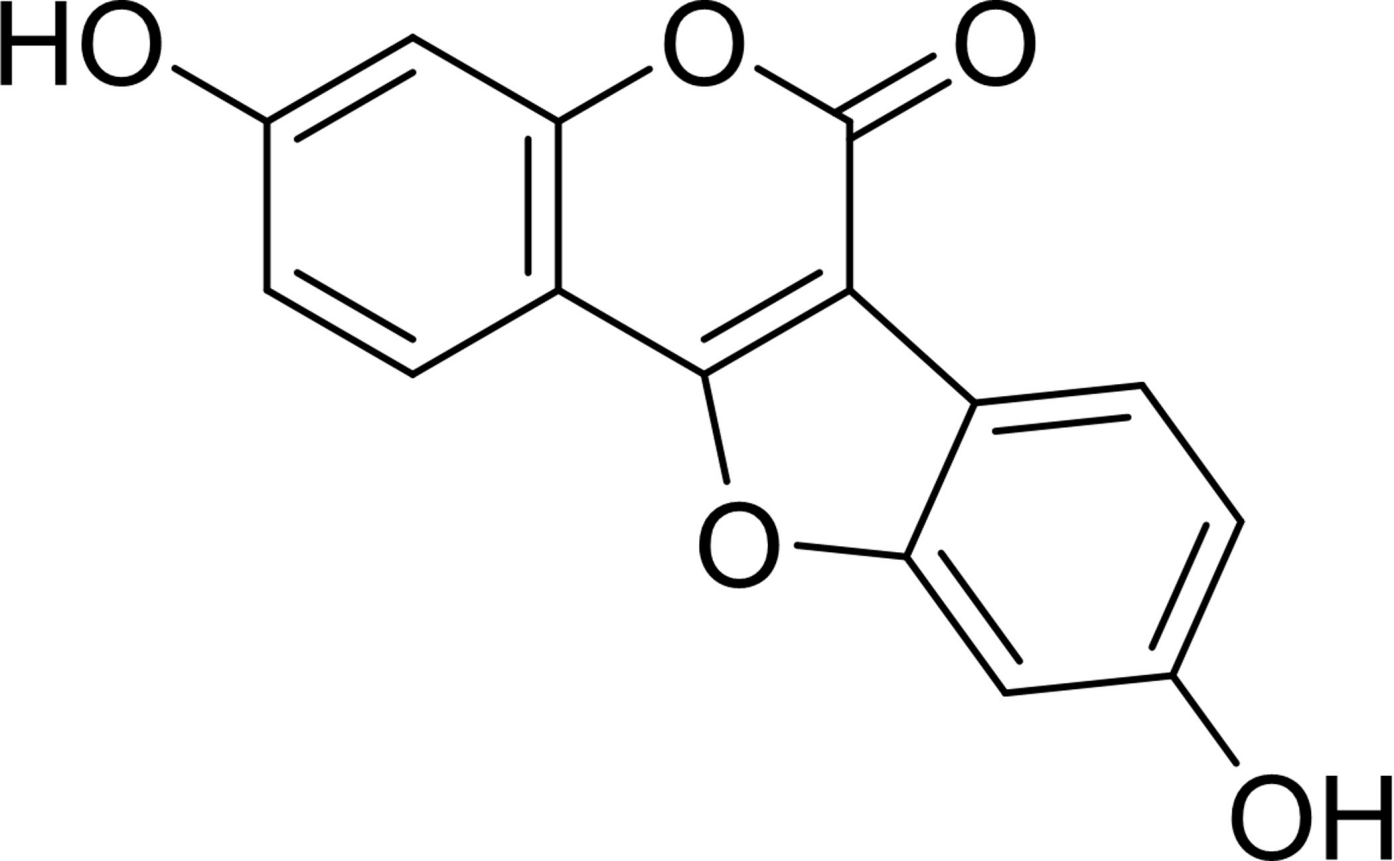
Chemical structure of CM.

Coumestrol (CM, Fig 1), a phytochemical in the class of coumestans, is a biologically active molecule. The CM biosynthesis pathway shares similarities with those of flavones and isoflavones [1]. CM is found mainly in *Leguminosae* plants such as red clover (*Trifolium pratense* Linnaeus) [2] and alfalfa (*Medicago sativa* Linnaeus) [3]. Among the vegetables on the market in Japan, CM has been identified in soybeans (*Glycine max* (L.) Merrill, seeds), soybean sprouts (*G*. *max* (L.) Merrill, sprouts), broccoli sprouts (*Brassica oleracea* var. *italica* Linnaeus, sprouts), Brussels sprouts (*B*. *oleracea* var. *gemmifera* de Candolle, sprouts), and pea sprouts (*Pisum sativum* Linnaeus, sprouts) [4–7]. CM’s biological activity may provide human health benefits through anticancer [8], anti-inflammatory [9], anti-Alzheimer’s disease [10] and anti-obesity [11,12] effects, as well as by inhibition of bone resorption [13]. A recent study determined that the anti-cancer effect of CM was due to direct targeting of HASPIN kinase, which phosphorylated of histone H3 at threonine 3 [8]. In addition, CM has been found to inhibit acetyl- and butyrylcholinesterase activity with IC_50_ values of 42.33 and 24.64 μM, respectively [10].

For the purpose of easily and efficiently increasing CM content in vegetables, the CM contents of eight vegetables commonly available in Japan were examined. Among them, soybean sprouts had the highest CM content.

Soybean sprouts are culinary vegetables grown by immersing soybean seeds in water and allowing them to germinate and grow in the dark. Soybean sprouts are extensively cultivated and consumed in Asian countries, including Japan, Korea, and China [14]. Among bean sprouts consumed in Japan, soybean sprouts have the highest nutritional value due to their high mineral, amino acid, protein, and dietary fiber contents [15]. Germinating soybeans to produce sprouts increases their aspartic acid, vitamin C, and total isoflavone contents [16,17]. Soybean sprouts include two organs, the cotyledon and hypocotyl, which contain different total amounts of isoflavones [18–20]. However, methods for quantifying and increasing CM content in soybean sprouts have not been reported.

In the present study, a new method for the quantitative analysis of CM was established to identify the vegetable with the highest CM content. Differences in CM content among the plant’s organs were also investigated. Furthermore, we examined whether CM content was affected by cultivation conditions such as light, water, and temperature. Our findings may lead to the development of novel approaches to increasing CM content in soybean sprouts.

## Materials and methods

### Plant materials and cultivation methods

The details of the plant materials and cultivation methods are shown in S1 Table. Voucher specimens were deposited at the Department of Pharmacognosy, Faculty of Pharmaceutical Sciences, Nagasaki International University, Japan.

#### CM extraction and HPLC analyses

GM-S soybean sprout hypocotyls from Oita, Japan, were used for these experiments. The samples were cultivated in a steel tray with tap water under artificial light for 1 day at 24°C and then lyophilized. Then the samples were pulverized before being subjected to HPLC analyses.

#### CM content in various vegetables

All vegetables were purchased from supermarkets in Nagasaki, Japan. The following nine vegetables were analyzed: broccoli (*B*. *oleracea* var. *italica* Linnaeus, flower buds) from Nagasaki, Japan; broccoli sprouts (*B*. *oleracea* var. *italica* Linnaeus, sprouts) from Fukuoka, Japan; *Brussels sprouts (B*. *oleracea* var. *gemmifera* de Candolle, sprouts) from Nagasaki, Japan; cabbage (*B*. *oleracea* var. *capitate* Linnaeus, leaves) from Nagasaki, Japan; Chinese cabbage (*B*. *rapa* Linnaeus var. *pekinensis* Ruprecht, leaves) from Miyazaki, Japan; pea sprouts (*P*. *sativum* Linnaeus, sprouts) from Fukuoka, Japan; soybeans (*G*. *max* (L.) Merrill, seeds) from Hokkaido, Japan; soybean sprouts (*G*. *max* (L.) Merrill, sprouts) from Oita, Japan; and white radish sprouts (*Raphanus sativus* (L.) var. *longipinnatus* L. H. Bailey, sprouts) from Fukuoka, Japan. All vegetable samples were lyophilized, pulverized, and subjected to HPLC analyses.

#### Difference in CM content among soybean sprout organs

Soybean sprouts from Oita, Japan, were divided into two organs, cotyledons and hypocotyls. The samples of the hypocotyls and cotyledons were lyophilized, pulverized, and then subjected to HPLC analyses.

#### Effects of temperature and light on CM content

Each sample consisted of 20 soybean sprouts cultivated in a tray (15 cm × 25 cm × 3 cm) containing tap water to a depth of about 5 mm. The water was changed twice a day. Two light conditions were used: one set of samples was provided with artificial light throughout the cultivation periods (“light”), and the other set was kept shaded during cultivation periods (“dark”). White fluorescent light (30W, 350 lux) was used as artificial light. Samples were kept in a temperature-controlled room at 24°C (room temperature) or 4°C (refrigerated) and were cultivated for 1, 2, or 4 days. After cultivation, samples were lyophilized, pulverized, and then subjected to HPLC analyses, as were uncultivated samples (0 days of cultivation).

#### Effects of bacteria and fungi on CM content

Soybean sprout hypocotyls from Oita, Japan, were cultivated under one of two water conditions: normal tap water (“water”) and tap water containing 1% antibiotic-antimycotic solution (penicillin-streptomycin-amphotericin B suspension, Wako Pure Chemical Industries, Ltd., Osaka, Japan) (“AFAB water”). Both water and AFAB water were changed twice a day. Samples were cultivated with water or AFAB water at 24°C in artificial light for 2 days before being lyophilized, pulverized, and subjected to HPLC analyses.

Identification of bacteria and fungi in soybean sprouts was performed by Japan Food Research Laboratories (JFRE, Osaka, Japan) following the guidelines of the Japanese Ministry of Health, Labor and Welfare in accordance with Standard Methods of Analysis in Food Safety Regulation [21,22].

#### Differences in CM content among commercial soybean sprouts

Soybean sprouts from Oita (Japan, product A), Nagano (Japan, product B and C), and Kyoto (Japan, product D) were lyophilized without any cultivation (“uncultivated”) or cultivated with tap water at 24°C in artificial light for 1 day and then lyophilized (“cultivated”). Then the samples were pulverized and subjected to HPLC analyses.

### HPLC sample solution preparation

For each sample, 50 mg dry pulverized powder was suspended in 1 mL methanol (MeOH, Nacalai Tesque Inc., Kyoto, Japan). Then the sample solution was heated to 50°C for 1 h for extraction. The extracts were centrifuged at 12,000 rpm for 10 min. Then the supernatant was filtered (Millex-LG, 0.20 μm, Merck KGaA, Darmstadt, Germany) and a 10 μL aliquot of the filtrate was analyzed using HPLC (*n* = 3).

### HPLC standard solution preparation

A 200 μg/mL standard stock solution was prepared by placing 2.00 mg CM (purity ≥ 95%; Merck KGaA, Darmstadt, Germany) in a volumetric flask and adding MeOH to the 10 mL mark. To prepare working solutions, aliquots of 0.5, 5, 50, and 500 μL the standard stock solution were transferred to volumetric flasks and diluted with MeOH to the 5 mL mark. The working solutions (200 μg/mL, 20 μg/mL, 2 μg/mL, 0.2 μg/mL, and 0.02 μg/mL) were used to construct calibration curves by injecting a 10 μL aliquot of each working solution into the HPLC system (*n* = 3).

### HPLC instrument and conditions

HPLC was performed using a JASCO LC-2000 Plus series instrument (JASCO, Tokyo, Japan) equipped with an intelligent UV-VIS detector (UV-2075 Plus), a quaternary gradient pump (PV-2089 Plus), an intelligent column oven (CO-2065 Plus), an intelligent autosampler (AS-2057 Plus), an LC-Net II/ADC user interface, and ChromNAV data analysis software. A COSMOSIL Cholester column (4.6 × 250 mm, particle size 5 μm, Nacalai Tesque INC. Kyoto, Japan) was used for HPLC analyses at 40°C. The mobile phase was a 40:60 (v/v) mixture of 0.1% formic acid (Wako Pure Chemical Industries, Ltd., Osaka, Japan) in acetonitrile (Nacalai Tesque Inc., Kyoto, Japan) and 0.1% formic acid in water. The flow rate was 1 mL/min, the injection volume was 10.0 μL, and the detection wavelength was 343 nm. The CM peak was observed at 11.1 min.

### Calibration, validation, and application of the established protocol

Calibration curves were constructed using five standards ranging in concentration from 0.02 to 200 μg/mL. Fresh calibration curves were prepared daily. The calibration curves were constructed by plotting concentration (μg/mL) on the horizontal axis and peak area (mV sec) on the vertical axis. Linearity was determined using the correlation coefficient (*R*^2^). GM-S extracts were used as matrix standards to determine the precision and accuracy of the analytical method. Intraday precision was determined by performing five injections using the same standard within 1 day. Interday precision was determined using injections of standards at the same concentration made over the course of 5 days. Recovery was determined by standard addition, in which 0.05 μg/mL CM was added to the sample solutions. CM content (μg/g, w/w) was calculated as the amount of CM (μg) divided by dry material weight (g).

### Statistical analyses

Each data point was calculated from at least three independent treatment repetitions. The results are expressed as the mean ± SD under each condition. The data were analyzed using GraphPad Prism 6 software (San Diego, CA, USA), and *p* < 0.001 was considered statistically significant. Statistical analysis was performed using Student’s t-test and one-way analysis of variance (ANOVA) with Dunnett’s or Tukey’s test. The statistical analysis methods used are described along with the results.

## Results and discussion

### CM extraction and HPLC analyses

First, we determined the conditions for the most efficient extraction of CM by comparing extraction efficacies between three solvent systems (water, 50% MeOH, and MeOH) under two conditions (heating at 50°C for 1 h or sonication for 1 h). The highest extraction efficiency (12.27 ± 0.11 μg/g CM content) was achieved by heating in MeOH (Table 1); thus, this condition was used for the remainder of the present study.

**Table 1:** CM content of soybean sprout hypocotyls extracted using three solvent systems (water, 50% MeOH, or MeOH) and two conditions (heating at 50°C for 1 h or sonication for 1 h).

Extraction method^a^	Solvent	CM content ^b^ (μg/g dry material)
Heating, 1 hour	MeOH	12.27 ± 0.11
	50% MeOH	1.27 ± 0.07*
	H_2_O	ND^c^
Sonication, 1 hour	MeOH	9.56 ± 0.24*
	50% MeOH	0.48 ± 0.11*
	H_2_O	ND^c^

^*a*^ Each extraction procedure was carried out once, using GM-S.

^*b*^ Values are means ± SD (*n* = 3). One-way analysis of variance (ANOVA) with Dunnett’s test was used to compare the group heated in MeOH for 1 h versus the other groups. The CM content in the group heated in MeOH for 1 h was significantly higher than in the other extraction method groups.

^*c*^ ND, not detected.

*p < 0.001.

**Fig 2:**
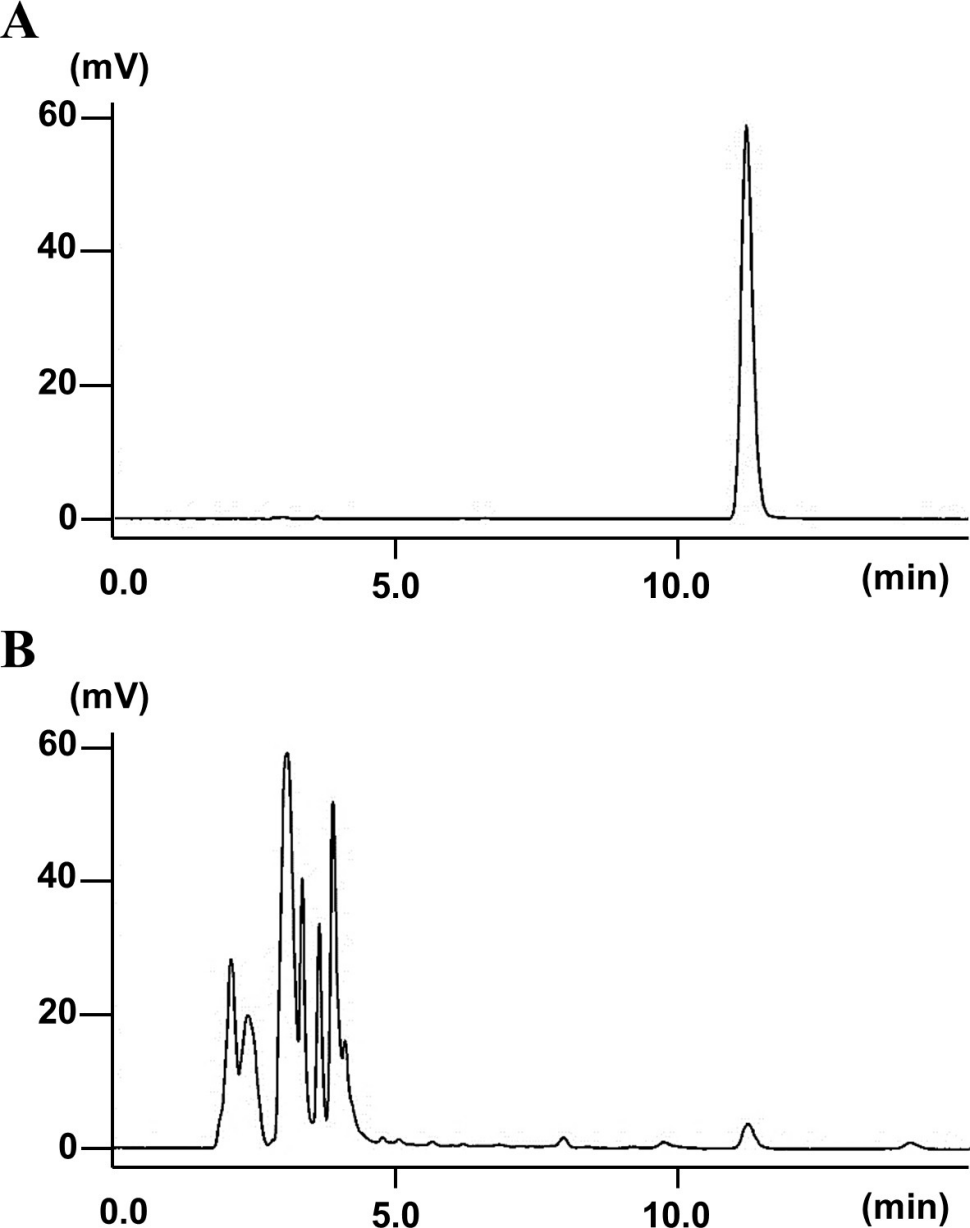
Typical HPLC chromatograms of a standard solution (20 μg/mL CM) (A) and MeOH extract of GM-S (50 mg/mL) (B).

Second, the CM content of the MeOH extract was determined using HPLC. Typical HPLC chromatograms for a standard and the GM-S extract are shown in Fig 2. The CM retention time was 11.1 min; the peak was identified by comparing the retention times with that of the standard.

Third, we evaluated linearity, intra- and interday precision, and accuracy (S2 Table). The linear regression equation for the calibration curve was *y* = 35.51*x*, where *x* is the concentration (μg/mL) and *y* is the peak area of the analyte (mV s). The calibration curve was linear over the range studied (*R*^2^ = 0.9999). Intraday and interday relative standard deviation (RSD) values using the GM-S extract were 1.53% and 2.54%, respectively. The accuracy was determined through recovery experiments using the GM-S extract. The mean recovery rate was 97.91% with an RSD of 1.87%. In sum, the analysis method was highly reproducible and accurate.

### CM content in various vegetables

**Fig 3:**
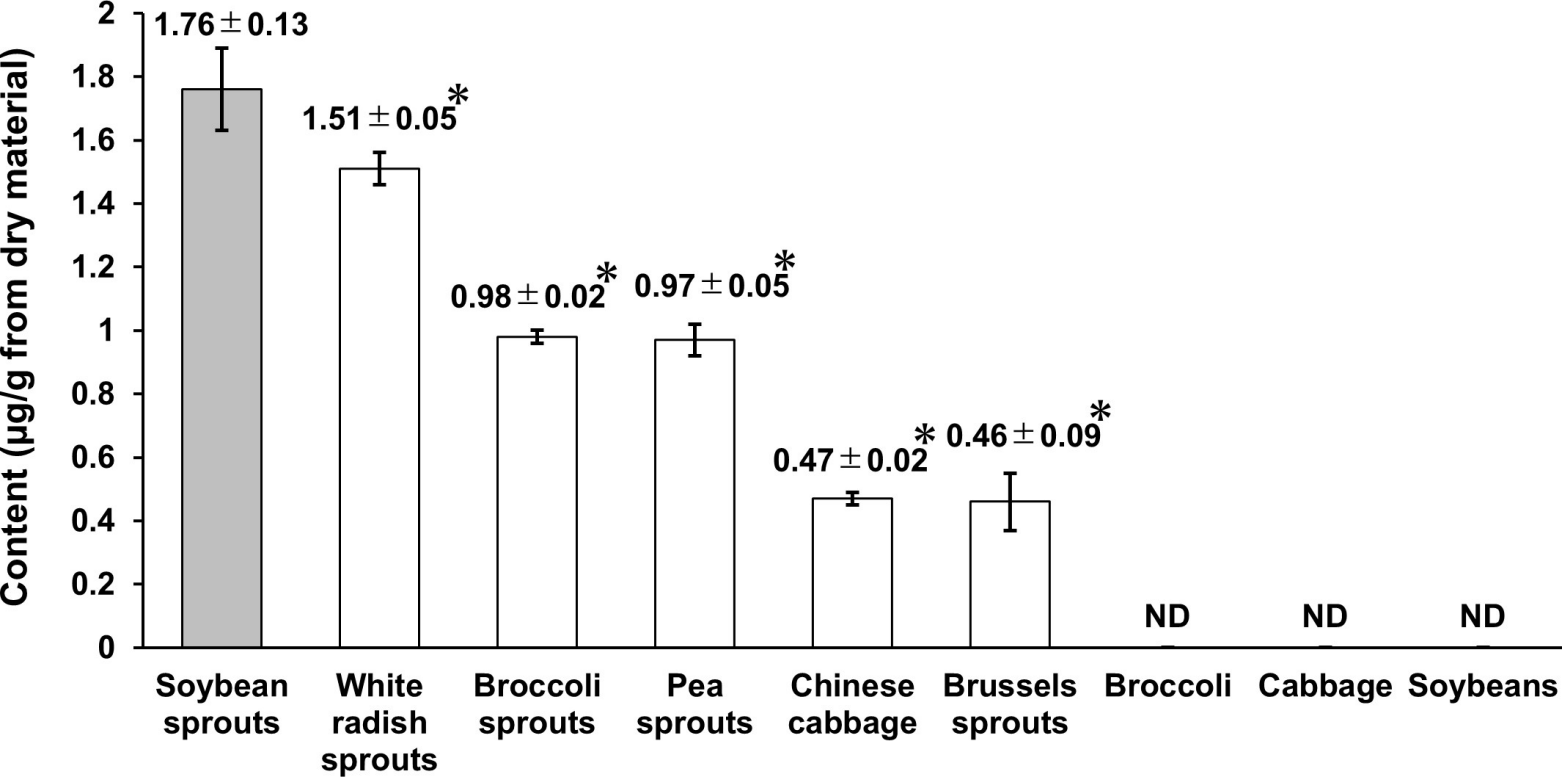
CM contents in nine vegetables (soybean sprouts, white radish sprouts, broccoli sprouts, pea sprouts, Chinese cabbage, Brussels sprouts, broccoli, cabbage, and soybeans). ANOVA with Dunnett’s test was used to compare the soybean sprout group to the other vegetable groups (*p < 0.001). The CM content was significantly higher in soybean sprouts than in the other vegetables. Values are means ± SD (*n* = 3). ND, not detected.

Using the method we established for CM extraction and HPLC analyses, vegetables with high CM content were examined. Of the vegetables that are readily available in Japan, nine were selected, either because they reportedly contained CM or because they belonged to the same genus or had the same organs as vegetables reported to contain CM. As shown in Fig 3, CM was detected in soybean sprouts (1.76 ± 0.13 μg/g), white radish sprouts (1.51 ± 0.05 μg/g), broccoli sprouts (0.98 ± 0.02 μg/g), pea sprouts (0.97 ± 0.05 μg/g), Chinese cabbage (0.47 ± 0.02 μg/g), and Brussels sprouts (0.46 ± 0.09 μg/g) but not in broccoli, cabbage, or soybeans. Because the CM content was significantly higher in soybean sprouts than in the other vegetables, soybean sprouts were used for the remainder of the present study.

Oshima *et al*. reported that CM was absent in ungerminated soybeans but was produced rapidly during germination [23], but they did not quantify soybean CM content before and after germination. By comparing CM content between soybean sprouts and soybeans, we showed that the CM content of soybeans was increased by germination.

### Difference in CM content among organs

**Fig 4:**
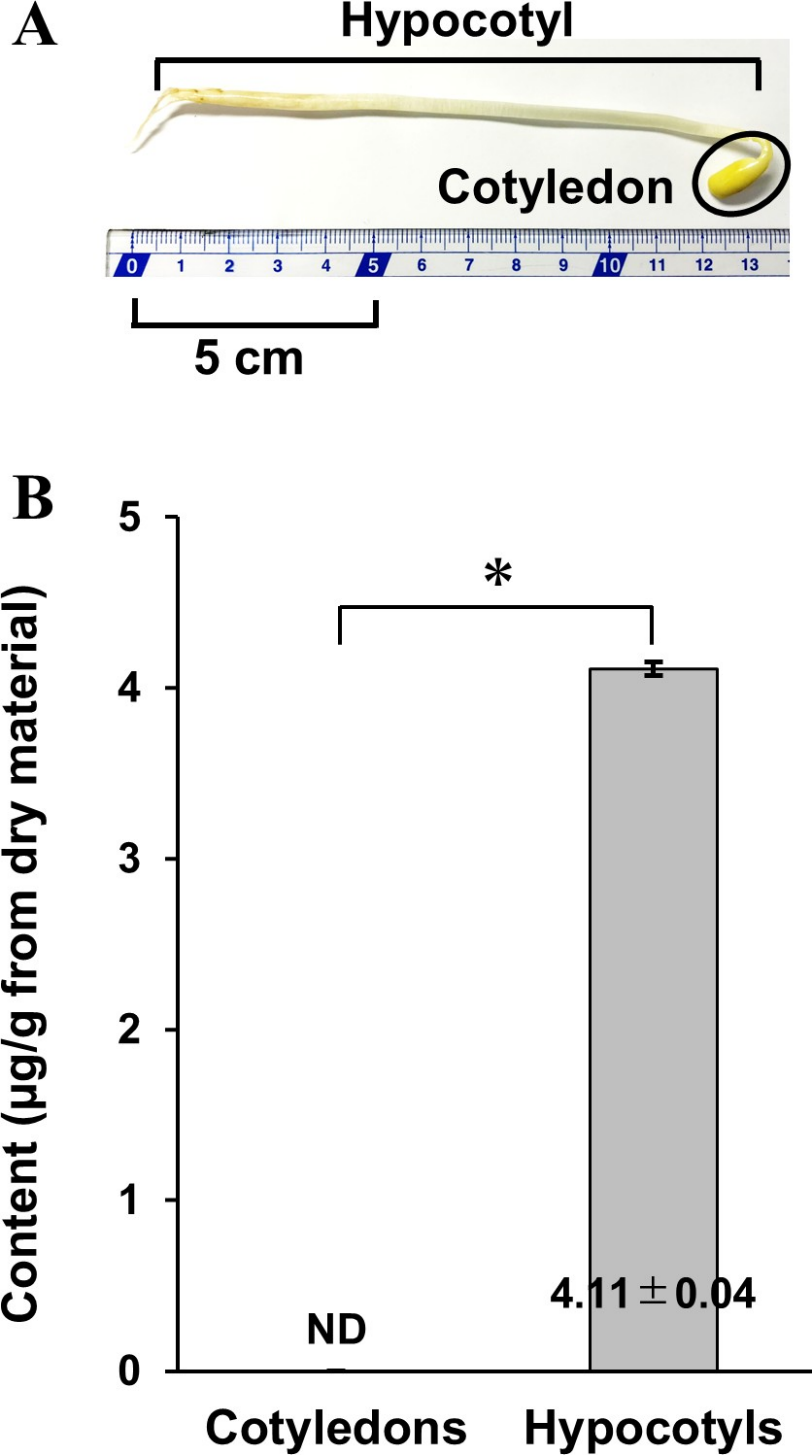
A soybean sprout (A) and CM contents in two soybean sprout organs, cotyledons and hypocotyls (B). The data were analyzed by Student’s t-test, and *p* < 0.001 (*) was considered statistically significant. Values are presented as mean ± SD (*n* = 3). ND, not detected.

We investigated the difference in CM content between two organs of the sprouts: the cotyledon and the hypocotyl (Fig 4A). As shown in Fig 4B, a CM content of 4.11 ± 0.04 μg/g was detected in hypocotyls, whereas CM was not detected in cotyledons.

### Effects of temperature and light on CM content

**Fig 5:**
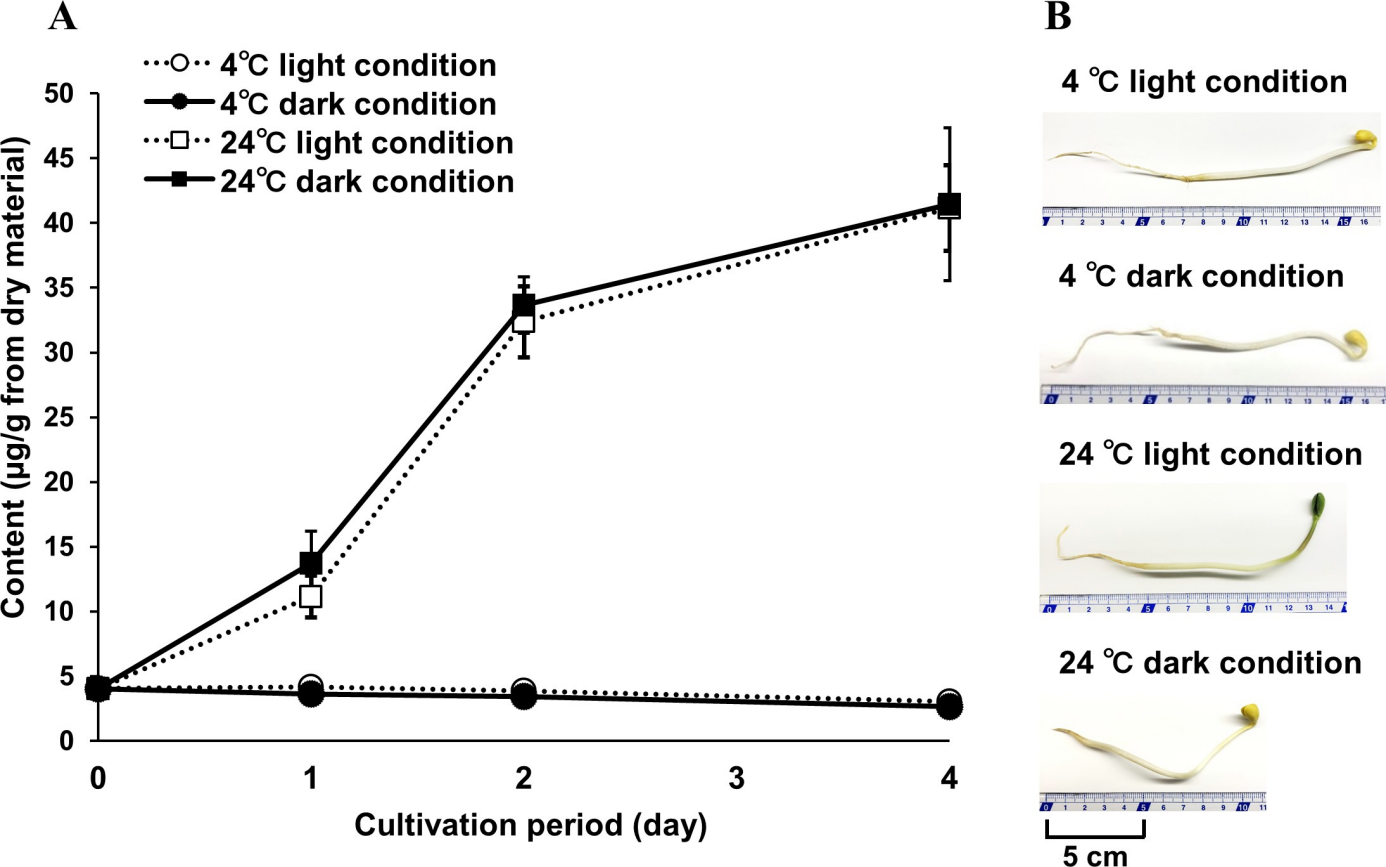
CM contents of soybean sprout hypocotyls grown under different light (light and dark) and temperature (4°C and 24°C) conditions (A), and soybean sprouts cultivated under each condition for 2 days (B). Sample details and CM contents are shown in Table 2. The data were analyzed by ANOVA followed by Tukey’s test, and *p* < 0.001 was considered statistically significant. There were no significant differences in CM content between the light and dark groups at 4°C or at 24°C. However, CM contents were significantly higher in the 24°C samples than in the 4°C samples in all cases (i.e., 4°C light versus 24°C dark, 4°C light versus 24°C light, and so forth).

We evaluated the CM contents of soybean sprouts cultivated under two temperature conditions (4°C and 24°C) and two light conditions (“light” and “dark”) (Table 2). Easily accessible temperatures—room temperature (24°C) and standard refrigerator temperature (4°C)—were selected because the goal of the study was to find a simple method to increase CM. The light conditions did not significantly affect the CM content of sprouts at either temperature (Fig 5A). However, the CM content was higher in sprouts cultivated at 24°C than in sprouts cultivated at 4°C, and the difference increased with increasing cultivation time: CM contents in the sprouts cultivated at room temperature were approximately 3, 9, and 14 times higher than in the refrigerated sprouts on days 1, 2, and 4, respectively. Taken together, these results demonstrate that temperature was most important in increasing CM content, while light conditions had little effect. Therefore, cultivation at 24°C is an effective method to induce CM production in sprouts.

**Table 2:** Sample details and CM contents of soybean sprout hypocotyls grown under different light (light and dark) and temperature (4°C and 24°C) conditions.

Sample name	Plant material		Cultivation methods				Content ^a^ (μg/g from dry material)
	Name	Organ	Water condition	Temperature condition (°C)	Light condition	Cultivation periods (days)	
4°C light condition	Soybean sprouts	Hypocotyls	Water	4	Light	0	4.05 ± 0.27
						1	4.18 ± 0.40
						2	3.86 ± 0.38
						4	3.03 ± 0.17
4°C dark condition					Dark	0	4.05 ± 0.27
						1	3.63 ± 0.28
						2	3.45 ± 0.32
						4	2.65 ± 0.73
24°C light condition				24	Light	0	4.05 ± 0.27
						1	11.15 ± 1.62
						2	32.37 ± 2.74
						4	41.14 ± 3.30
24°C dark condition					Dark	0	4.05 ± 0.27
						1	13.72 ± 2.47
						2	33.67 ± 2.17
						4	41.43 ± 5.90

^*a*^ Values are means ± SD (*n* = 3).

### Effects of fungi and bacteria on CM

Boué *et al*. reported that *Aspergillus* species induced CM production in soybeans, leading to accumulation of CM [24]. To examine the effects of fungi and bacteria in our cultivation methods, we compared the CM contents of samples prepared using normal tap water (“water”) and tap water obtained antifungal and antibacterial agents (“AFAB water”). As shown in S1 Fig, there were no differences in CM content between the water (11.3 ± 0.95 μg/g) and AFAB water (11.4 ± 0.39 μg/g) samples. In addition, growth of bacteria and fungi was observed in soybean sprouts cultivated with tap water but not in those cultivated with AFAB water. In addition, the bacteria and fungi in soybean sprouts cultured in water were investigated. Two types of enteric bacteria and three types of non-fermenting gram-negative bacilli were isolated from the soybean sprouts. The total number of bacteria was 3.5 × 10^7^/g, similar to that in typical soybean sprouts [25,26]. Furthermore, the total number of molds was 70 /g and that of yeasts was 7.8 × 10^2^ /g, similar to that in typical soybean sprouts [27]. These data suggest that the growth of bacteria and fungi had little effect on the cultivation methods used here.

### Differences in CM content among commercial soybean sprouts

**Fig 6:**
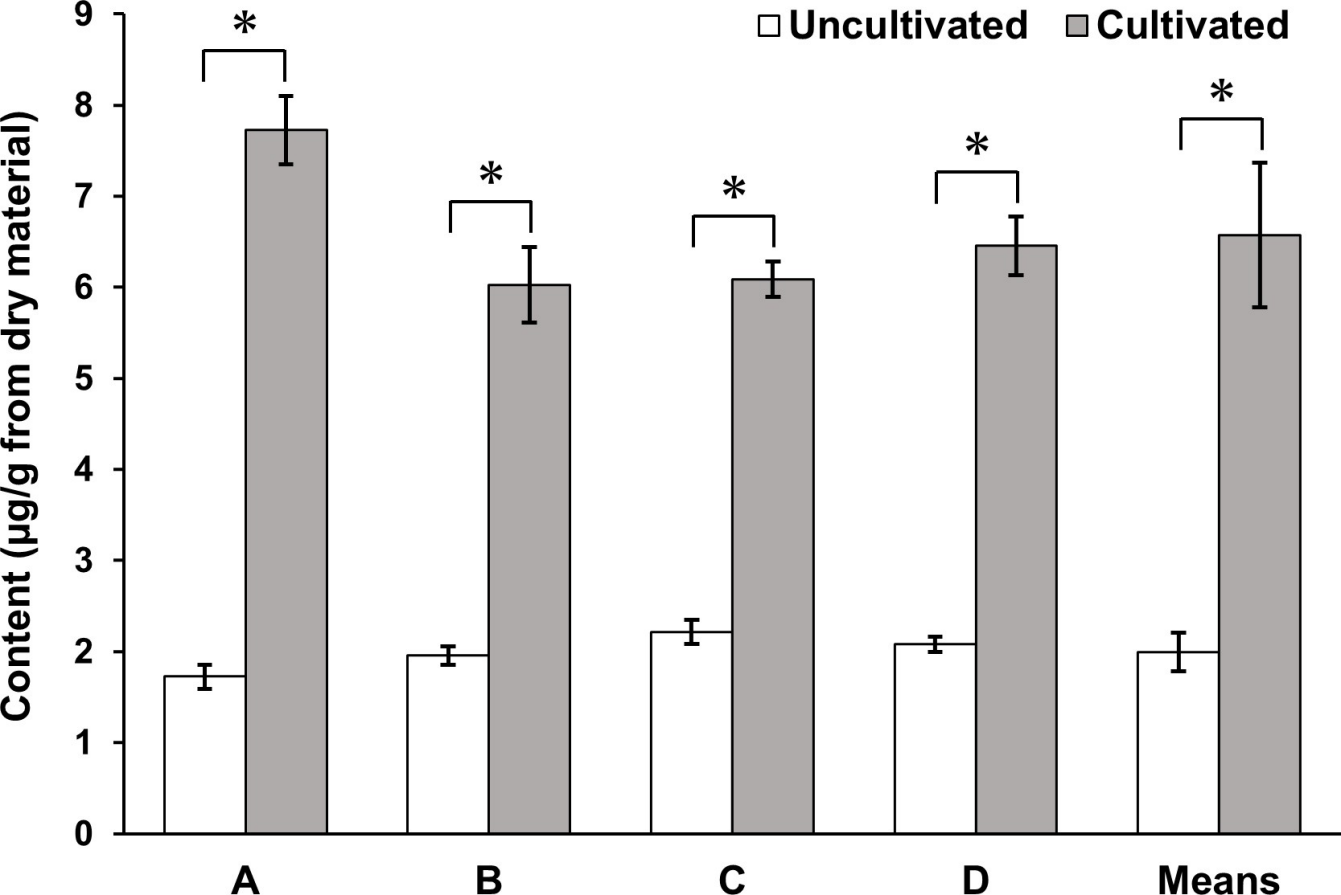
CM content in various commercial soybean sprouts. Sample details and CM contents are shown in Table 3. Four soybean sprouts (products A–D) were used as samples. Samples were either cultivated at 24°C in light for 1 day and then lyophilized (“cultivated”) or lyophilized immediately (“uncultivated”). Uncultivated and cultivated groups were compared using Student’s *t*-test, and *p* < 0.001 was considered statistically significant. CM content was significantly higher in the cultivated groups than in the uncultivated groups; mean CM contents were 6.57 ± 0.79 and 2.00 ± 0.21 μg/g dry material in the cultivated and uncultivated groups, respectively.

To identify differences in CM content among commercial soybean sprouts, the CM contents of four types of soybean sprouts (products A–D) were examined without cultivation (“uncultivated”) or with cultivation at 24°C in light for 1 day (“cultivated”). Cultivation at 24°C increased CM content by 3.3 times on average in all soybean sprouts (Fig 6 and Table 3), demonstrating that our established methods are applicable to commercial soybean sprouts.

**Table 3:** Sample details and CM contents in various commercial soybean sprouts.

Sample name		Plant material			Cultivation methods				Content ^b^ (μg/g dry material)
		Name	Organ	Source	Water condition	Light condition	Temperature condition (°C)	Cultivation periods (days)	
A	Uncultivated	Soybean sprouts	Sprouts	Oita, Japan	– ^a^				1.73±0.13
	Cultivated				Water	Light	24	1	7.72±0.37
B	Uncultivated			Nagano, Japan	– ^a^				1.96±0.10
	Cultivated				Water	Light	24	1	6.02±0.41
C	Uncultivated				– ^a^				2.22±0.14
	Cultivated				Water	Light	24	1	6.09±0.19
D	Uncultivated			Kyoto, Japan	– ^a^				2.08±0.08
	Cultivated				Water	Light	24	1	6.46±0.32

^*a*^ Samples were lyophilized without being cultivated.

^*b*^ Values are means ± SD (*n* = 3).

Several studies have reported a relationship between soybean sprout color and constituents, with higher nutrients such as protein, fat, vitamins, and minerals in green sprouts than in yellow sprouts [28]. Chi *et al*. reported that green sprouts accumulated more total isoflavones than yellow sprouts [29]. However, Lee *et al*. reported no differences in total isoflavone content between green and yellow sprouts [18]. We therefore also investigated the differences in CM content between sprout colors. As shown in Fig 5B, cultivating the soybeans at 24°C in the light produced green sprouts, while yellow sprouts were produced under all other cultivation conditions. However, there were no differences in CM content between green (light conditions) and yellow (dark conditions) sprouts cultivated at 24°C. Thus, we concluded that CM content could not be predicted based on sprout color.

The maximum tolerable daily intake of CM for humans is estimated at 22 μg/kg body weight [30,31]. This value was calculated from the lowest observed adverse effect level in an animal study [32]. Due to the low content of CM in foods, daily intake is typically considered to be negligible. In this study, the quantity of CM in 1 g of dry weight of soybean sprouts was about 41 μg using our cultivation method. Because the amount of soybean sprouts per bag was about 200 g and more than 90% of soybean sprouts are water, the dry weight of soybean sprouts per bag was less than 20 g. When soybean sprouts were cultivated using our method, the maximum CM content per bag was 820 μg. From the above, a soybean sprout bag cultivated using our method does not exceed the upper limit of the tolerable daily intake of adults, and so CM intake will have a beneficial effect on health.

## Conclusions

We developed an HPLC method for qualitative analyses of CM in vegetables and investigated which cultivation conditions produced soybean sprouts with high CM contents. We found that soybean sprouts had the highest CM content of the nine vegetables investigated, that CM was not present in soybean seeds and was only produced after sprout germination, and that more CM accumulated in the hypocotyls than in the cotyledons. Finally, we found that CM content depended on temperature but not on light condition, the growth of fungi and bacteria, or sprout color. Our findings verified methods for increasing CM content in soybean sprouts easily and efficiently, which is valuable given the known beneficial biological activity of CM [9–14]. These results represent progress in cultivation methods of soybeans sprouts and their use for promoting human health.

## Supporting information

S1 Fig CM contents of soybean sprout hypocotyls grown under different water conditions (water and AFAB water). Sample details are shown in the table. The data were compared using Student’s t-test, and *p* < 0.001 (*) was considered statistically significant. Values are means ± SD (*n* = 3). There were no significant differences in CM content between samples with water and those with AFAB water.
(TIF)

S1 Table Plant materials and cultivation methods for each section. ^*a*^ Samples were continuously provided with artificial light during cultivation periods. ^*b*^ Samples were kept shaded during cultivation periods. ^*c*^ Room temperature. ^*d*^ Temperature in the general refrigerator. ^*e*^ Samples were given tap water twice a day. ^*f*^ Samples were given tap water with 1% antibiotic-antimycotic solution twice a day. ^*g*^ Samples after cultivation were lyophilized and subjected to HPLC. ^*h*^ Samples without any cultivation were lyophilized and subjected to HPLC.
(PDF)

S2 Table Linearity, range, precision, and recovery for determination of CM content in soybean sprout hypocotyls. ^*a*^ In the regression equation, *x* is the concentration of the analyte solution (μg/mL), and *y* is the peak area of the analyte (mV sec). ^*b*^ Precision of the analytical method was tested using MeOH extracts of GM-S (*n* = 5). ^*c*^ Recoveries spiked with MeOH extracts of GM-S. ^*d*^ Values are means ± RSD (*n* = 5).
(PDF)
