# Supplementary figures and images for: Effective methods for increasing coumestrol in soybean sprouts
Source: PLoS One. 2021 Nov 18;16(11):e0260147. doi: 10.1371/journal.pone.0260147 (PMC8601530; doi:10.1371/journal.pone.0260147)

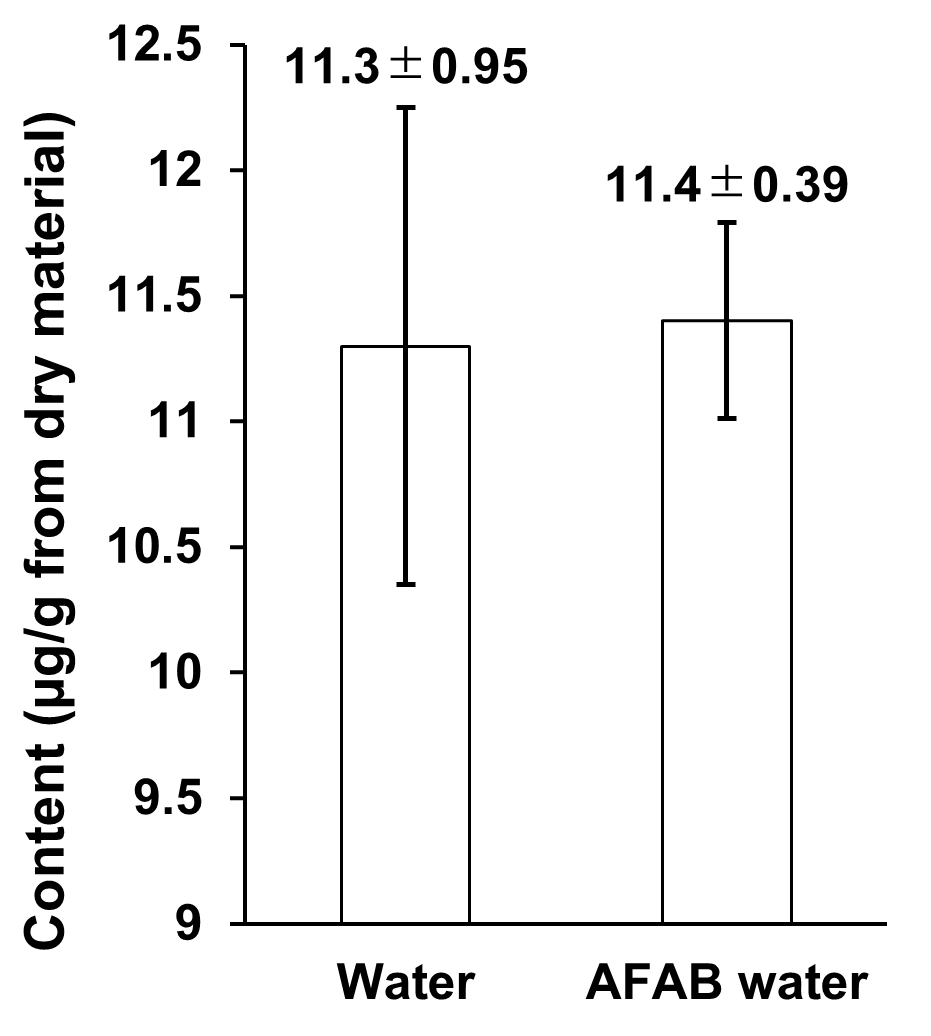

Supplement: S1 Fig — Sample details are shown in the table. The data were compared using Student’s t-test, and p < 0.001 (*) was considered statistically significant. Values are means ± SD (n = 3). There were no significant differences in CM content between samples with water and those with AFAB water. (TIF) [file pone.0260147.s001.tif]
