# Supplementary material for: Effective methods for increasing coumestrol in soybean sprouts
Source: PLoS One. 2021 Nov 18;16(11):e0260147. doi: 10.1371/journal.pone.0260147 (PMC8601530; doi:10.1371/journal.pone.0260147)
Supplement: S1 Table — a Samples were continuously provided with artificial light during cultivation periods. b Samples were kept shaded during cultivation periods. c Room temperature. d Temperature in the general refrigerator. e Samples were given tap water twice a day. f Samples were given tap water with 1% antibiotic-antimycotic solution twice a day. g Samples after cultivation were lyophilized and subjected to HPLC. h Samples without any cultivation were lyophilized and subjected to HPLC. (PDF) [file pone.0260147.s002.pdf]

S1 Table. Plant materials and cultivation methods for each section.

Section	Sample name		Plant material			Cultivation methods			
			Name	Organ	Source	Light condition	Temperature condition (°C)	Water condition	Cultivation periods (days)
CM Extraction and HPLC analysis (Table 1, S2 Table, and Fig.2)	GM-S		Soybean sprouts	Hypocotyls	Oita, Japan	Light[a]	24[c]	Water[e]	1[g]
CM content in various vegetables (Fig. 3)	Broccoli		Broccoli	Flower buds	Nagasaki, Japan	–[h]	–[h]	–[h]	–[h]
	Broccoli sprouts		Broccoli sprouts	Sprouts	Fukuoka, Japan	–[h]	–[h]	–[h]	–[h]
	Brussels sprouts		Brussels sprouts	Axillary buds	Nagasaki, Japan	–[h]	–[h]	–[h]	–[h]
	Cabbage		Cabbage	Leaves	Nagasaki, Japan	–[h]	–[h]	–[h]	–[h]
	Chinese cabbage		Chinese cabbage	Leaves	Miyazaki, Japan	–[h]	–[h]	–[h]	–[h]
	Pea sprouts		Pea sprouts	Sprouts	Fukuoka, Japan	–[h]	–[h]	–[h]	–[h]
	Soybeans		Soybeans	Seeds	Hokkaido, Japan	–[h]	–[h]	–[h]	–[h]
	Soybean sprouts		Soybean sprouts	Sprouts	Oita, Japan	–[h]	–[h]	–[h]	–[h]
	White radish sprouts		White radish sprouts	Sprouts	Fukuoka, Japan	–[h]	–[h]	–[h]	–[h]
Difference in CM content among organ (Fig. 4)	Hypocotyls		Soybean sprouts	Hypocotyls	Oita, Japan	–[h]	–[h]	–[h]	–[h]
	Cotyledons		Soybean sprouts	Cotyledons	Oita, Japan	–[h]	–[h]	–[h]	–[h]
Effects of temperature and light on CM content (Fig. 5 and Table 2)	4°C light condition		Soybean sprouts	Hypocotyls	Oita, Japan	Light[a]	4[d]	Water[e]	0~4[g]
	4°C dark condition		Soybean sprouts	Hypocotyls	Oita, Japan	Dark[b]	4[d]	Water[e]	0~4[g]
	24°C light condition		Soybean sprouts	Hypocotyls	Oita, Japan	Light[a]	24[c]	Water[e]	0~4[g]
	24°C dark condition		Soybean sprouts	Hypocotyls	Oita, Japan	Dark[b]	24[c]	Water[e]	0~4[g]
Effects of bacteria and fungi on CM content (S1Fig.)	Water		Soybean sprouts	Hypocotyls	Oita, Japan	Light[a]	24[c]	Water[e]	2[g]
	AFAB water		Soybean sprouts	Hypocotyls	Oita, Japan	Light[a]	24[c]	AFAB water[f]	2[g]
Differences in CM content among commercial soybean sprouts (Fig. 6 and Table 3)	A	Uncultivated	Soybean sprouts	Sprouts	Oita, Japan	–[h]	–[h]	–[h]	–[h]
		Cultivated	Soybean sprouts	Sprouts	Oita, Japan	Light[a]	24[c]	Water[e]	1[g]
	B	Uncultivated	Soybean sprouts	Sprouts	Nagano, Japan	–[h]	–[h]	–[h]	–[h]
		Cultivated	Soybean sprouts	Sprouts	Nagano, Japan	Light[a]	24[c]	Water[e]	1[g]
	C	Uncultivated	Soybean sprouts	Sprouts	Nagano, Japan	–[h]	–[h]	–[h]	–[h]
		Cultivated	Soybean sprouts	Sprouts	Nagano, Japan	Light[a]	24[c]	Water[e]	1[g]
	D	Uncultivated	Soybean sprouts	Sprouts	Kyoto, Japan	–[h]	–[h]	–[h]	–[h]
		Cultivated	Soybean sprouts	Sprouts	Kyoto, Japan	Light[a]	24[c]	Water[e]	1[g]

[a]Samples were continuously provided with artificial light during cultivation periods. [b]Samples were kept shaded during cultivation periods. [c]Room temperature. [d]Temperature in the general refrigerator. [e]Samples were given tap water twice a day. [f]Samples were given tap water with 1% antibiotic-antimycotic solution twice a day. [g]Samples after cultivation were lyophilized and subjected to HPLC. [h]Samples without any cultivation were lyophilized and subjected to HPLC.
