# Supplementary material for: Effective methods for increasing coumestrol in soybean sprouts
Source: PLoS One. 2021 Nov 18;16(11):e0260147. doi: 10.1371/journal.pone.0260147 (PMC8601530; doi:10.1371/journal.pone.0260147)
Supplement: S2 Table — a In the regression equation, x is the concentration of the analyte solution (μg/mL), and y is the peak area of the analyte (mV sec). b Precision of the analytical method was tested using MeOH extracts of GM-S (n = 5). c Recoveries spiked with MeOH extracts of GM-S. d Values are means ± RSD (n = 5). (PDF) [file pone.0260147.s003.pdf]

S2 Table. Linearity, range, precision, and recovery for determination of CM content in soybean sprout hypocotyls.

Regression equation[a]	Correlation coefficient	Range (μg/ml)	Precision[b] (RSD, %)		Recovery[c, d] (RSD, %)
			Intraday	Interday	
$y = 35.51x$	0.9999	0.02 – 200	1.53	2.54	97.91 ± 1.87

[a]In the regression equation, x is the concentration of the analyte solution (µg/mL), and y is the peak area of the analyte (mV sec).

[b]Precision of the analytical method was tested using MeOH extracts of GM-S ($n = 5$).

[c]Recoveries spiked with MeOH extracts of GM-S. [d]Values are presented as mean ± RSD ($n = 5$).
